# Puzzling obstructive tumor in the right heart: A large intravascular leiomyomatosis with intracardiac extension

**DOI:** 10.21542/gcsp.2025.9

**Published:** 2025-02-28

**Authors:** Marha M. Menaisy, Ahmed Esmail, Ahmed Ramadan, Omar Dawoud, Ahmad Samir

**Affiliations:** 1 Cardiology Department, Kasr AlAiny School of Medicine, Cairo University, Cairo, Egypt; 2 Radiology Department, Kasr AlAiny School of Medicine, Cairo University, Cairo, Egypt; 3 Department of Cardio-thoracic Surgery, Kasr AlAiny School of Medicine, Cairo University, Cairo, Egypt; 4 Cardiology Department, Cardiac Center Hail, Hail, Saudi Arabia

## Abstract

Objective: Intravascular leiomyomatosis (IVL) is a rare benign tumor capable of hematogenous spread. Clinical presentation is heterogeneous depending on the destination organ, which often delays identification.

Key steps: A 38-year-old female with a recent history of hysterectomy presented with dyspnea and dizziness. Initial workup revealed a huge obstructive growth in the right heart chambers. A thrombus-in-transit was the most likely preliminary diagnosis. Further thorough assessment reduced the likelihood of a thrombus and increased suspicion of a benign tumor. A large IVL with intracardiac extension was then considered. The mass was completely resected through a combined one-stage approach, and IVL was confirmed pathologically. The patient was well-educated about the importance of periodic surveillance for the reported high recurrence rates.

What we have learned? IVL is a rare benign tumor that behaves like malignancy and, hence may have nonspecific presentations. Thorough clinical evaluation and collaborated multi-modality imaging are critical keys to the appropriate diagnosis.

## Introduction

Intravascular leiomyomatosis (IVL) is a rare smooth muscle cell tumor that although benign, often spreads hematogenously reaching remote sites^1^. Commonly, IVL originates from the smooth muscles of uterine fibroid tumors (hence termed leiomyomatosis), although in rare case reports may arise from gastro-intestinal tract muscles^2^. The clinical manifestations of IVL are non-specific and mostly related to the mass effect at the involved site, often resulting in delayed diagnosis or accidental identification of large IVLs by imaging^1,3^. Management of IVL is always challenging, because of the multisite involvement and the high recurrence rate^4^. Cardiac extension occurs in approximately 20–30% of IVL cases and often presents with nonspecific symptoms. Clinical manifestations can vary widely, ranging from dyspnea, palpitations, and chest tightness to being entirely asymptomatic, while in some cases, sudden cardiac death can be the first presentation^3,5^.

In this report, we present a case of a middle-aged female with progressive dyspnea and dizziness who was discovered to have a huge obliterative intracardiac mass. Although a thrombus in transit or a metastasizing malignancy were the most likely scenarios, considering the patient’s background, the multi-disciplinary approach, and the multi-modality imaging guided the appropriate diagnosis and management of a rare IVL with obstructive intracardiac extension. 

## Case presentation

A 38-year-old female presented complaining of progressive dyspnea, palpitation, and dizziness for 2 months. She had a single 7-year-old child and no significant medical history, apart from a uterine fibroid excision 3 years prior, followed by a hysterectomy and right oophorectomy 8 months before the current presentation. Pathological examination confirmed a benign leiomyoma. The patient reported having secondary amenorrhea since then, also she had not received any hormonal replacement therapies.

**Figure 1.**
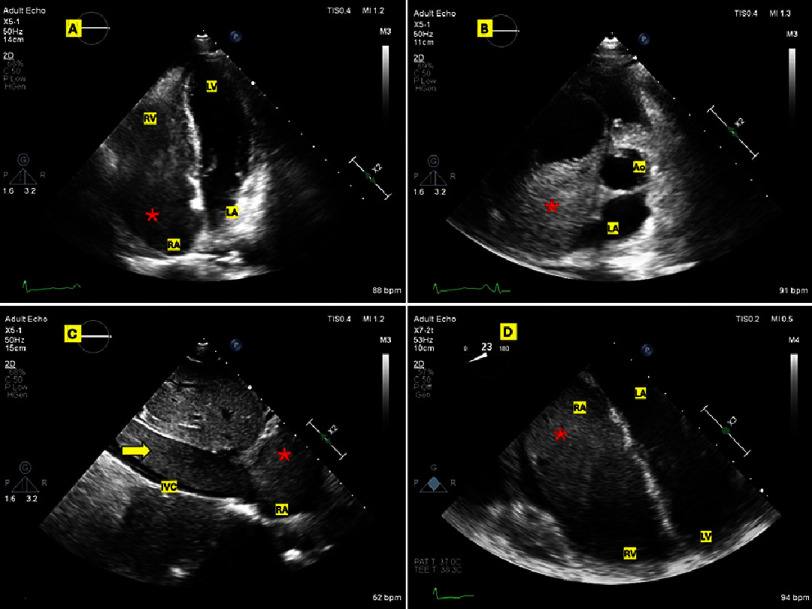
Pre-operative TTE and TEE. (A): TTE A4C view showing the large mass (red asterisk) occupying RA and RV. (B) SAX view with the mass prolapsing from RA to RV. (C) The elongated mass in IVC (red arrow) entering RA. (D) TEE 4-chamber view with the mass nearly filling RA and RV. A4C, Apical 4-chamber; IVC, Inferior vena cava; RA, Right atrium; RV, Right ventricle; SAX, Short axis; TEE, Transesophageal echocardiography; TTE, Transthoracic echocardiography.

Upon evaluation, the patient’s physical examination was unremarkable, except for mild ankle edema and elevated jugular venous pulsations. However, the transthoracic and transesophageal echocardiography (TTE and TEE) revealed a large mass almost completely obstructing the right atrium (RA) and right ventricle (RV). The mass had well-defined, regular margins, was mostly not adherent to the endocardium, and was mobile through the cardiac cycle. It was predominantly solid, with a cystic appearance in the part hanging in the RV apex and appeared to be continuous with a subocclusive extension in the inferior vena cava (IVC). (Figure 1) (Supplemental videos 1–3).

**Figure 2.**
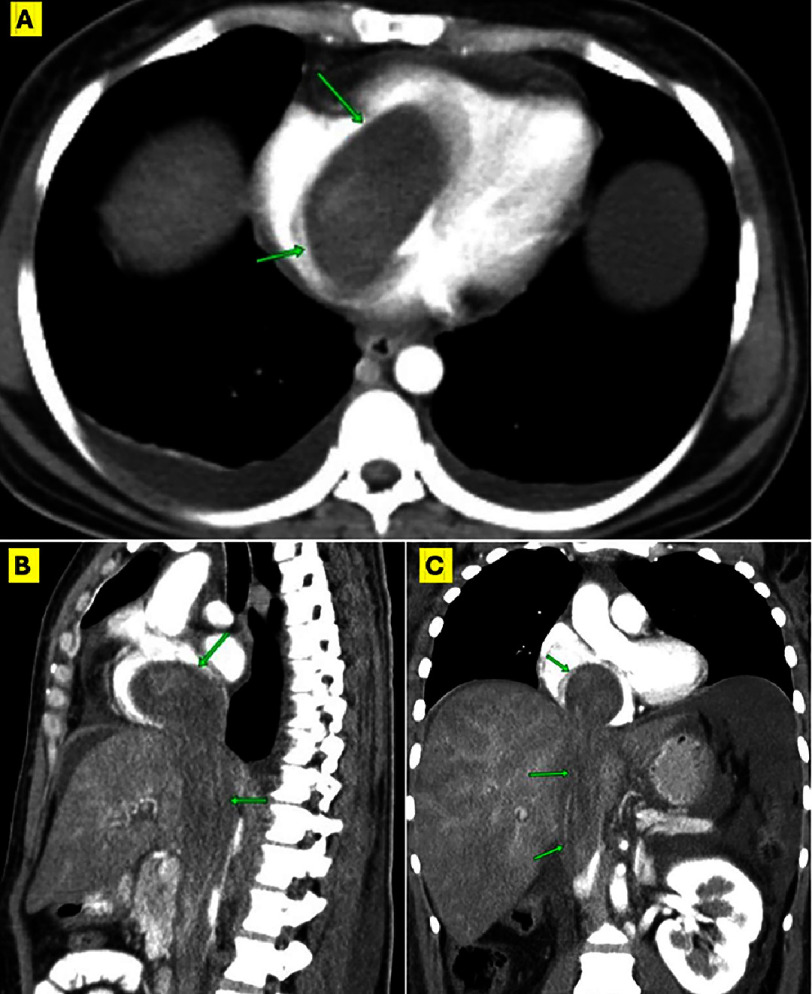
Pre-operative thoracoabdominal CT with contrast. (A) Contrast-enhanced CT showing a 4-chamber view and the large mass (green arrows) prolapsing from the RA to the RV. (B) Sagittal- and (C) coronal CT views showing the mass extending from the IVC to the RA. CT, Computed tomography; Other abbreviations as in Figure 1.

The initial impression was a deep venous thrombosis (DVT) with a large thrombus in transit or metastatic malignancy, while a primary cardiac tumor was the next possibility. Accordingly, therapeutic anticoagulation was initiated until further workup ensued. Ultrasonographic examinations revealed total occlusion of the right internal and common iliac veins and the infrarenal segment of the IVC, while the rest of the abdominal and thoracic IVC was distended by the floating, hyperechoic mass extending proximally. Enhanced computed tomography (CT) confirmed the large soft tissue mass distending the inferior vena cava and extending into the right heart chambers with heterogeneous enhancement. (Figure 2).

**Figure 3.**
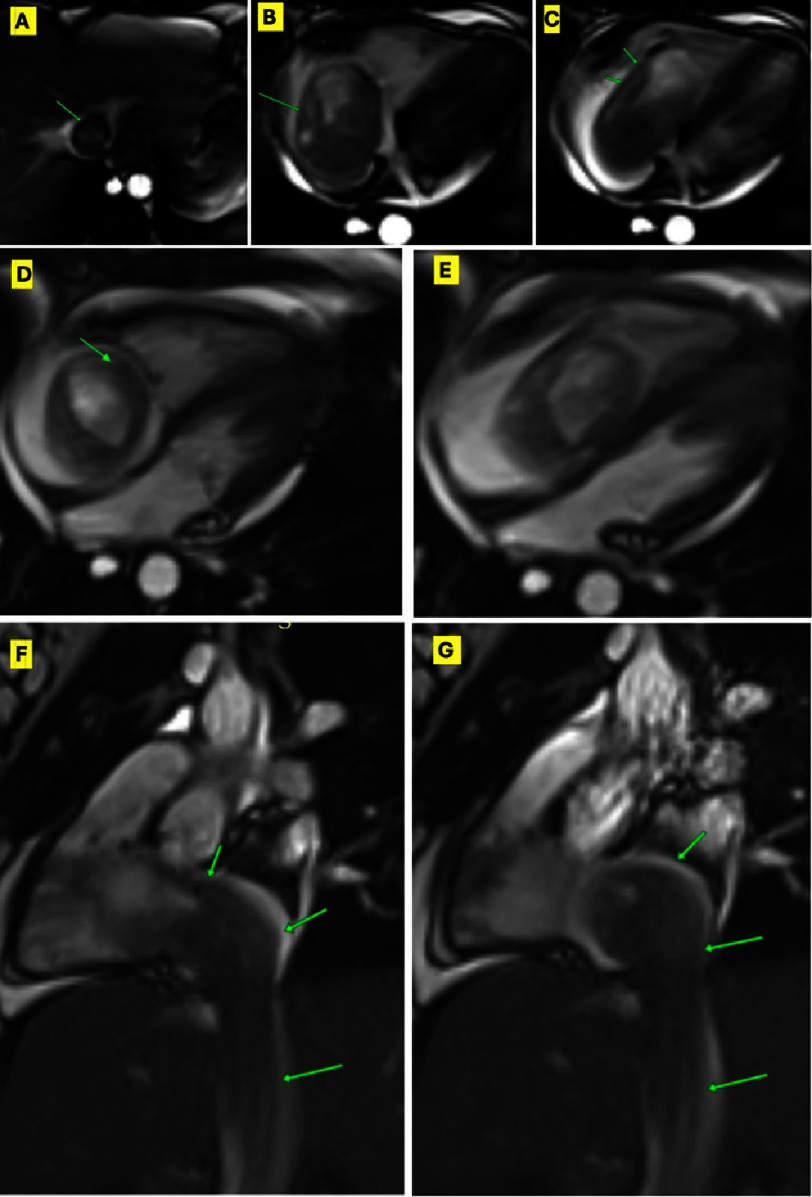
Pre-operative CMR. (A), (B), and (C) showing CMR SSFP images of the mass (green arrow) in the IVC, RA, and RV, respectively. (D) and (E) showing SSFP 4-chamber view in systole and diastole respectively demonstrating the mass prolapse into RV in diastole. (F) and (G) showing the elongated extending from IVC to RA. CMR, Cardiac magnetic resonance; SSFP, Steady state free precession; Other abbreviations as in Figure 1.

**Figure 4.**
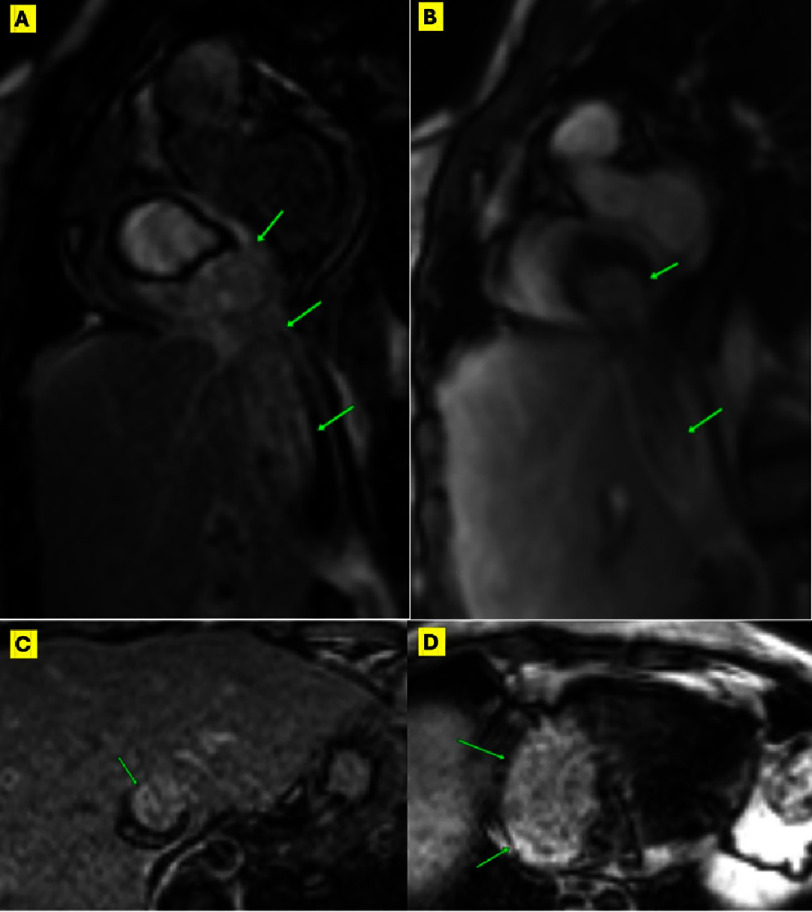
Pre-operative Post-contrast CMR. Post-contrast CMR sequences demonstrating the intense early perfusion of the mass (in A) and the intense delayed hyper-enhancement (in B) denoting neoplastic nature. (C) and (D) Axial post-contrast inversion recovery, demonstrating the intense delayed hyper-enhancement of the mass in IVC and RA respectively, supporting a neoplastic nature. Green arrows point to the mass in the corresponding panels. Abbreviations as in Figure 3.

Although the diagnosis of DVT was very plausible, we believed that the history of recurrent uterine fibroids should warrant further investigations. In cardiac magnetic resonance (CMR) post-contrast sequences, the mass demonstrated intense early perfusion and delayed hyperenhancement, while demonstrating delayed hyper-enhancement in the post-contrast inversion recovery, suggesting a neoplastic nature. The lack of invasion of contiguous structures was in favor of a benign lesion. (Figures 3, 4) (Supplemental videos 4–6).

Thus, a large intravenous leiomyomatosis extending to the heart had to be considered, despite the absence of detectable pelvic masses and the several months period after hysterectomy. The patient underwent a combined, single-stage approach for complete tumor resection. The resection was performed through median-sternotomy, cardiopulmonary bypass, right atriotomy, and pulling the IVC growth.

**Figure 5.**
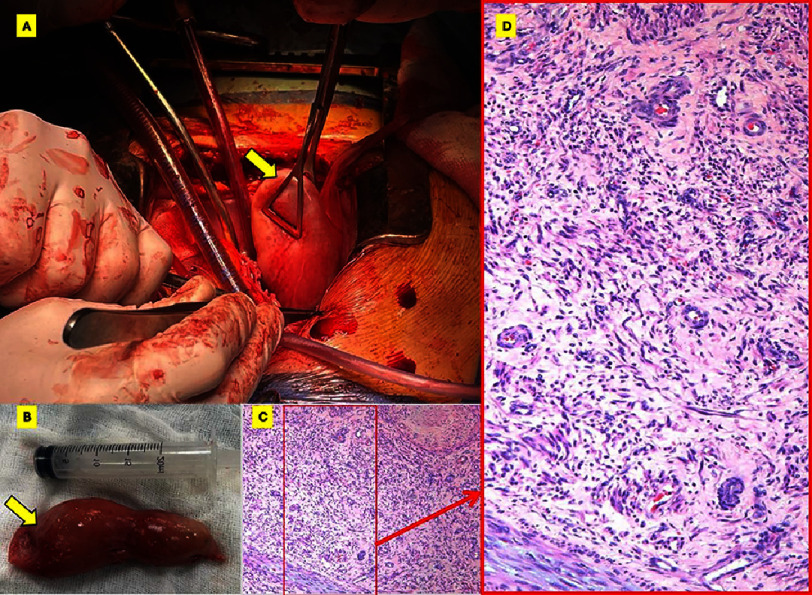
Intra-operative images and histopathological assessment. (A) Intraoperative image demonstrating grasping the mass (yellow arrow) through the RA incision. (B) The resected intracardiac extension of the mass. (C) Microscopic examination [Hematoxylin and Eosin, x100] showing the proliferating smooth muscle bundles, with blunt-ended nuclei, and eosinophilic cytoplasm characteristic of leiomyomatosis. (D) A digital zooming of the red box marked in (C) to visualize the smooth muscles’ cellular proliferation.

The operative specimen, comprising the intracardiac and intracaval components, was 25 cm in length and weighed 190 grams. The histopathological assessment revealed uninterrupted proliferation of spindle-shaped smooth muscle cells with no signs of atypia or infiltration to neighboring tissues, confirming the diagnosis of leiomyomatosis (Figure 5).

**Figure 6.**
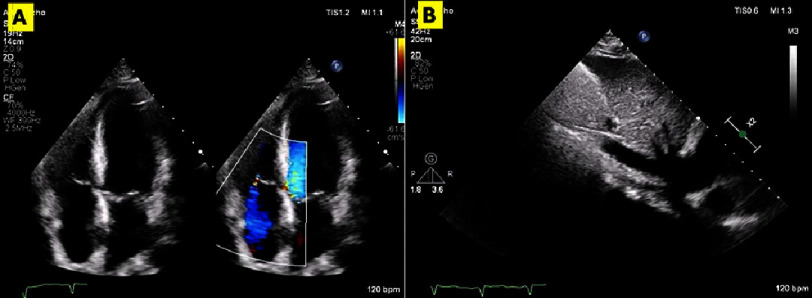
Post-operative TTE. Post-operative TTE showing: (A) A4C view after mass resection and only mild tricuspid regurgitation by color; (B) IVC in subcostal view after complete mass resection in contrast to Figure 1.

**Figure 7.**
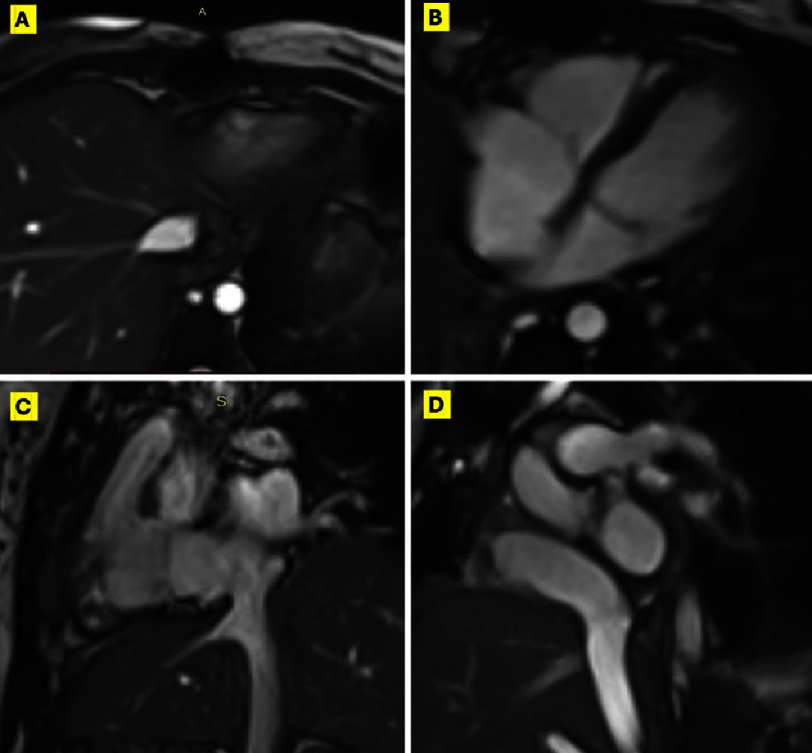
Post-operative CMR. Post-operative CMR SSFP axial images on the IVC (in A), 4-chambers view (in B) as well as coronal (in C) and sagittal (in D) demonstrating complete removal of the mass in contrast to the pre-operative images in Figure 4. Abbreviations as in Figure 3.

The patient had an uneventful postoperative course and was discharged on the 7th day. Follow-up TTE and CMR one month postoperatively showed normal ventricular function and no residual masses. (Figures 6, 7) (Supplemental videos 7–10). After discussion with the oncology team, a short course of 3-month hormonal therapy was planned to reduce the risk of recurrence. The patient was well-educated about her condition and the importance of periodic surveillance with planned repeat imaging every 3 months for the first year, then bi-annually. The timeline management of this case is visually represented in Figure 8.

## Discussion

Histologically, IVL is a benign tumor that originates from smooth muscle cells typically of uterine fibroids^1^. The origin of IVL is presumed to be a cellular nidus from the uterine/para-uterine smooth muscles invading the draining blood vessels. Although pathologically benign, IVL behaves like malignancy by spreading through the vascular system to remote sites and may develop large tumorous masses in distant organs^2,3^. Unlike malignant metastases, IVL grows (like a worm) within the vascular tubes, scarcely forming adhesions or leading to complete obstruction^3,4^.

Typically, IVL develops in the 40s and 50s of menstruating parous females^3,6^. The exact incidence of IVL is unclear, however, it ranges between 0.25%-to-0.41% in uterine leiomyomas reports, while in a recently published series of IVL cases, 71% had sizable uterine fibroids and 90% had prior obstetric/gynecological surgeries^1,4^.

The diagnosis of IVL is often delayed because of the non-specific presentation. Occasionally It may be discovered incidentally during imaging workups for various manifestations, depending on the affected site, size, and extent of the tumor growth^1^. In cases with cardiac involvement, reported presentations varied from asymptomatic, exertional dyspnea, heart failure, or serious complications like syncope or sudden death, likely due to RV outflow obstruction or massive pulmonary embolism^1,3,4^.

Starting with a large cardiac mass, the diagnosis and management are often challenging. The common differential diagnoses include thrombi in transit, secondaries of malignant tumors, and primary cardiac tumors, while other rare aetiologies represent minorities^7^. Although IVL is an extremely rare cause of cardiac tumors, a thorough analysis of the patient’s history (underscoring uterine bleedings, previous fibroids, or hysterectomy), may warrant considering IVL as a possible diagnosis.

In this case, presenting with exertional dyspnea as the chief complaint, the history of a relatively recent surgery, the total occlusion of the right iliac vein, and the IVC with a mass extending to the right heart chambers; all prioritized the preliminary diagnosis of a large thrombus-in-transit. Furthermore, having a hysterectomy 8 months prior and the absence of any primary pelvic masses (peri-uterine or adnexal) could have inclined the thoughts away from IVL.

Nevertheless, we favored a non-thrombotic etiology from the TTE picture (a wormy growth from the IVC to the heart that is non-adherent to the endocardium). Also, in light of the patient’s history of recurrent fibroids, a late-developing IVL was regarded as a differential diagnosis. Hence, CMR was performed and was quite helpful in advocating a non-thrombotic, mostly benign tumor lesion, while the definite diagnosis of IVL was clinched by the histopathological examination of the resected mass. Although very rare, IVL developing months to years after hysterectomy had been previously reported^8^.

The curative management of IVL mandates complete resection of the primary tumor (pelvic mass) and extraction of the intravascular tumor growth^3,8,9^. There is no settled predilection in intracardiac IVLs for a single-stage or two-stage surgical approach. The single-stage approach entails median sternotomy, cardiopulmonary bypass, and right atriotomy to extract the intracardiac growth followed by pulling and resection of the IVC growth through a concomitant laparotomy access. The two-stage approach entails a dedicated session for sternotomy and then laparotomy in a separate subsequent session. The prolonged cardiopulmonary bypass and the heparinization with possible excess bleeding are the cons, yet a single exposure to anesthesia and minimizing chances of tumor spillage are the pros for the single-stage over the two-stage approach^3,9^.

Recurrence rates of IVL can be as high as 30% within the first year after surgery, yet with favorable overall survival rates^3,4^. Incomplete tumor resection and age <45y were identified as significant predictors for IVL recurrence^3,4,8^. Although IVL typically possesses Estrogen and Progesterone receptors and are considered hormone-sensitive tumors, experiences with hormonal therapies as an alternative to surgical resection are disappointing, and their current use is mainly to minimize recurrence or slow down the growth of unresectable extensions^10,11^.

## What have we learned?

Intracardiac extension of leiomyomatosis is considered an extremely rare scenario. When the intracardiac growth is the first to draw medical attention, the diagnosis of IVL can be puzzling and requires a high index of clinical suspicion. Multi-modality imaging, multi-disciplinary, and ensuring complete tumor resection are crucial for appropriate diagnosis and management of IVL.

Extended periodic surveillance is needed acknowledging the high rates of recurrence.

## Videos legends

Video S1. Apical 4-chamber view in transthoracic echocardiography revealing the large mass occupying the right atrium (RA) and right ventricle (RV) https://youtu.be/E2Y6I9i-QJU.

Video S2. Parasternal short-axis view demonstrating the large mass filling the RA cavity and prolapsing in diastole to the RV https://youtu.be/0j080g5sdrQ.

Video S3. Subcostal view showing the elongated “nearly occlusive” growth of the mass in the inferior vena cava (IVC) extending to the RA. https://youtu.be/mh5duQhSKG8.

Video S4. Mid-esophageal 4-chamber view by trans-esophageal echocardiography with the large mass occupying the RA and RV. https://youtu.be/YJwLP5baNP4.

Video S5. Cardiac magnetic resonance (CMR) pre-operative cine axial view on the chest and abdomen showing large IVC mass extending to the RA, obstructing the tricuspid valve, prolapsing into the RV during diastole. https://youtu.be/Vc51Ijm8GtM?si=EA2djEQkU15Ycwsl.

Video S6. CMR pre-operative cine four chambers view showing large IVC mass extending from the RA through the tricuspid valve into the RV. https://youtu.be/PKWixYYqNRw?si=iffOu_WQAEZJ213u.

Video S7. CMR pre-operative cine sagittal images on the IVC showing the large IVC mass extending to the RA and RV. https://youtu.be/QnHPJmd-mgY?si=YGlbCijjxL62nuOd.

Video S8. CMR pre-operative cine short-axis view showing large IVC mass extending to the RA. https://youtu.be/ll2bnCE8Sos?si=5zlYdcDlr9ygIjPr.

Video S9. CMR Dynamic post contrast images on the IVC mass showing rapid early intense post contrast enhancement suggesting neoplastic nature. https://youtu.be/OxAmjtL2py8?si=fEdmyvJPzAZUbGnk.

Video S10. Postoperative transthoracic echocardiography showing apical 4 chamber with color compare views. It demonstrates the complete resection of the mass and no more than mild tricuspid regurgitation. https://youtu.be/0CiK1FyamVg.

Video S11. Postoperative transthoracic echocardiography demonstrating a subcostal view for the inferior vena cava after the resection of the mass. https://youtu.be/oj8tCDzcrns.

Video S12. CMR post operative four chambers cine view showing total mass resection. https://youtu.be/bTCc9RUlaco?si=xz5eHAYK7krMiDvQ.

Video S13. CMR post operative coronal view on the IVC showing a patent IVC after complete mass resection. https://youtu.be/y8TuDOm4MB0?si=5X1m5WBFLcQ-mRR8.

Video S14. CMR post operative sagittal view on the IVC showing a patent IVC after complete mass resection. https://youtu.be/4sz0F5AQ5No?si=5mZVtC8GujmdqTzW.
